# Effects of Oral Administration of *Lactiplantibacillus Plantarum* APsulloc 331261 (GTB1^TM^) Isolated from Green Tea on Atopic Dermatitis (AD)-like Skin Lesion Mouse Models

**DOI:** 10.1155/2022/4520433

**Published:** 2022-09-06

**Authors:** Su-Young Kim, Jung Ok Lee, Yu-Jin Kim, You Na Jang, Jung Min Lee, A. Yeon Park, Kwang-Ho Yoo, Beom Joon Kim

**Affiliations:** ^1^Department of Dermatology, College of Medicine, Chung-Ang University, Seoul 06974, Republic of Korea; ^2^Department of Medicine, Graduate School, Chung-Ang University, Seoul 06973, Republic of Korea

## Abstract

**Background:**

Probiotics are known to improve atopic dermatitis (AD) by inhibiting T helper 2 (Th2)-related reactions, restoring the Th2/T helper1 (Th1) cytokine ratio. The most popular probiotic is *Lactiplantibacillus plantarum* (*L. plantarum*), which is widely used in the food and pharmaceutical industries. *L. plantarum* APsulloc 331261 (GTB1) used in this study was isolated from green tea.

**Materials and Methods:**

The effectiveness of oral GTB1 administration in improving AD was evaluated by visual evaluation, comparison of the lymph node sizes and spleen weights, histological evaluation, RT-qPCR, ELISA, and IHC analysis in the mouse model.

**Results:**

GTB1 improved AD symptoms, reduced epidermal thickness and mast cell numbers, decreased lymph node size and the spleen weight, increased filaggrin and loricrin protein levels, downregulated Th2 expression, and upregulated Th1 expression in a colony-forming unit-dependent manner.

**Conclusion:**

Oral administration of GTB1 isolated from green tea (*Camellia sinensis*) improved the AD symptoms, reduced hypersensitivity reaction, and increased the skin barrier function. Finally, it is involved in AD improvement by restoring the Th2/Th1 cytokine balance.

## 1. Introduction

Atopic dermatitis (AD) is a chronic inflammatory skin disease with symptoms including eczematous lesions, impaired epidermal barrier, pruritus, and dry skin [1]. The exact cause of AD is unknown. A combination of immune dysregulation, epidermal gene mutations, stress, and environmental factors are known to be involved. In an immunological viewpoint, an imbalance between CD4^+^ T helper type1 (Th1) and CD4^+^ T helper type2 (Th2) cell-mediated responses is the main cause of AD [2,3]. By exposing the skin to various exogenous antigens, interleukin-4 (IL-4), IL-5, and IL-13 express more than IL-2 and interferon gamma (IFN- *γ*) to strengthen the humoral immune response resulting in a profound overexpression of Th2-related cytokines [4].

Generally, to suppress inflammation and itching, steroids, antihistamines, cyclosporine, and methotrexate are used as therapeutics [5]. However, these drugs cause side effects such as hypertension and liver and kidney toxicity with long-term treatment [6]. As another treatment, inhibition of the JAK signaling pathways is a way to reduce the activation of multiple proinflammatory mediators involved in the pathogenesis of AD [7, 8]. Although there are variety treatments, for the non-responders to conventional therapies, new biological drugs with few adverse effects and high effectiveness are consistently being demanded.

Probiotics are defined as living microorganisms that give the host health benefits when administered in an appropriate amount [9]. Functionally, probiotics can regulate the microecological balance of the digestive tract, inhibit the growth and adhesion of pathogenic bacteria, stimulate epithelial cell proliferation, and ameliorate inflammation resulting in a healthy digestive tract [10, 11]. Furthermore, probiotics restore the imbalance between Th1 and Th2 by inhibiting Th2-related reactions [12].

*Lactiplantibacillus* and *Bifidobacterium* are the most commercialized probiotic strains [13]. Among them, *Lactiplantibacillus plantarum* (*L. plantarum*) is widely used in the food and pharmaceutical industries [14] and is effective in alleviating the clinical symptoms of AD in children [15, 16]. *L. plantarum* APsulloc 331261 (GTB1) is isolated from green tea (*Camellia sinensis*) which has more adhesion to intestinal epithelial surfaces than another *Lactobacillus* spp and has an advantage to the stomach and gut by increasing the number of beneficial bacteria [17, 18]. According to the previous study, gut microbiome changes skin phenotypes with AD patients by affecting the immune responses in the skin [19]. Taken all together, we hypothesized that GTB1 may ameliorate AD-like lesions caused by gut-skin axis. Therefore, in this study, we examined the effects of GTB1 on oxazolone (4-ethoxymethylene-2-phenyl-2-oxazolin-5-one, OXZ)-induced AD-like lesions in hairless mice.

## 2. Materials and Methods

### 2.1. Animals and Animal Experimental Design (Groups 1-5)

**Figure 1:**
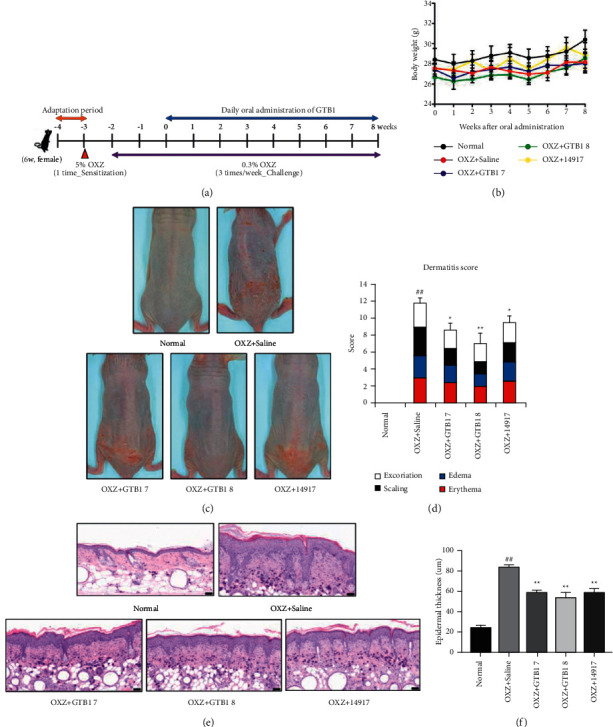
Design of the entire experiment (a) and body weight changes (b) over 8 weeks of study. Representative macroscopic photography of mouse dorsal skin in each administration group at 8 weeks (c). The dermatitis score was analyzed by summing signs shown in AD-like dorsal skin lesions i.e., excoriation, scaling, edema, and erythema, with scores of 0 (clear), 1 (almost clear), 2 (mild), 3 (moderate), and 4 (severe) for each sign (d) (*n* = 5/group). Histological examination of dorsal skin lesion using H&E staining (20*X*, Scale bar = 50 *μ*m) (e). Epidermal thickness was measured by counting three spots in each group (f). OXZ + GTB1 7, GTB1 1 × 10^7^ CFU per mouse; OXZ + GTB1 8, GTB1 1 × 10^8^ CFU per mouse; OXZ + 14917, 14917 1 × 10^8^ CFU per mouse. Results are expressed as the mean ± SEM (*n* = 3/group). ^*∗*^*p* < 0.05, ^*∗∗*^*p* < 0.01 vs OXZ + Saline; ^##^*p* < 0.01 vs Normal.

Six-week-old female hairless mice were purchased from Saeron Bio Inc. (Gyeonggi-do, South Korea) and provided sufficient solid feed (antibiotic-free) and water until the day of the experiment. They were adapted to a temperature of 23 ± 2°C, a humidity of 55 ± 10%, and a light-dark cycle for 1 week before being used in the experiments. All animal testing processes were approved by the Chung-Ang University Animal Laboratory Ethics Committee (202000047) and the applicable National Institutes of Health guidelines were followed. The normal group (Group 1) received no treatment. OXZ + Saline group (Group 2) received saline only. OXZ + GTB1 7 (Group 3) group received 1 × 10^7^ CFU GTB1 per mouse. OXZ + GTB1 8 (Group 4) group received 1 × 10^8^ CFU GTB1 per mouse. OXZ + 14917 (Group 5) group received 1 × 10^8^ CFU 14917 per mouse. Three weeks after the application of OXZ, all groups (*n* = 5/group) were administered with the drugs daily for 8 weeks using a syringe and a feeding needle catheter (Figure 1(a)).

### 2.2. Preparation of Probiotics

GTB1 (KCCM11179 P, GTB1^TM^) used in this study was isolated from green tea (Osulloc Farm, Jeju Island, South Korea) and provided by AMOREPACIFIC (Yongin, South Korea). The stability characteristics of this probiotic have been described previously [17]. *Lactiplantibacillus plantarum* 14917 (14917) was used as type strain and purchased from the American Type Culture Collection. GTB1 and 14917 were cultured in Lactobacilli de Man Rogosa Sharpe broth (MRS; Becton, Dickinson and Company; Sparks, MD, USA) at 37°C for 18 h. CFU/mL corresponding to the OD_600_ value of 1 was calculated before animal testing for cultured *Lactiplantibacillus plantarum* strains, which was confirmed by plating a serial diluent on the MRS agar plate. Two strains pellet, respectively, were obtained by centrifugation (12,000 g for 5 min at 4°C). The pellet was washed twice with phosphate-buffered saline (pH 7.4) and adjusted to colony-forming unit (CFU) in 200 *µ*l of saline according to the oral administration groups.

### 2.3. Induction of AD-like Lesions (Sensitization and Challenge)

The induction of AD in hairless mice using OXZ was performed according to the method described by Man et al. [20]. To sensitize the mice, the skin at the back of the neck of all mice, except for those in the normal group, was treated with 10 *µ*l of 5% OXZ (Sigma-Aldrich, USA) dissolved in acetone and olive oil (at a 4 : 1 ratio) at −3 weeks (Sensitization step). After 1 week, the lower part of the back of the mice was treated with 60 *µ*l of 0.3% OXZ, 3 times a week, for a total of 10 weeks to induce AD (Challenge step).

### 2.4. Measurement of Dermatitis Severity

Anestheic (Zoletil + Rompun: 0.008 cc/10 g (40 mg/kg) + 0.002 cc/10 g (5 mg/kg)) was dilute (10-fold) with normal saline, and the dorsal skin of anesthetized test animals was photographed in proximity using a digital single-lense reflex camera (D5200; Nikon, Tokyo, Japan). According to the method described by Kang et al. [21], the severity of AD-like dorsal skin lesions was assessed by dermatitis score at the end of the experimental period. The degree of each symptom, such as excoriation, scaling, edema, and erythema was scored as 0 (almost clear), 1 (mild), 2 (moderate), and 3 (severe). Clinical skin score, defined as the sum of the individual scores, ranged from 0 to 12. Dermatitis severity was evaluated by 2 independent observers.

### 2.5. Body Weight, Spleen Weight, and Lymph Node Size

The body weight was measured weekly. Changes in spleen weights were measured using electronic scale. Changes of the inguinal lymph nodes size were measured using a ruler at the end of the experiment after treatment. Changes in spleen weight were normalized to each body weight.

### 2.6. Histological Analysis

To investigate the effect of the GTB1 on AD, the lesion area of the test animal tissue was fixed in 10% neutral buffered formalin at the end of the experiment. After step-by-step dehydration using a high-concentration ethanol solution (starting from a low concentration), paraffin blocks were prepared. Each tissue block was cut to a thickness of 5 *μ*m to facilitate attachment to the slide, and each tissue slide was deparaffinized with xylene and dehydrated using alcohol. To evaluate epidermal thickening, tissue slides were stained with hematoxylin and eosin (H&E). Mast cells in the skin were stained with toluidine blue (TB). Some skin sections were stained for anti-filaggrin (1 : 250, GTX37695, Gene Tex) and anti-loricrin (1 : 2000, ab85679, Abcam) antibodies in accordance with the manufacturer's instructions. The slices were washed with PBS, dehydrated, and mounted in a Permount mounting medium (SP15-100, Thermo Scientific). All stained tissue slides were photographed using a slide scanner (Pannoramic MIDI; 3DHISTECH Ltd, Budapest, Hungary) and observed using Case Viewer software. All histological examinations were analyzed in 3 sections/animal slices.

### 2.7. Transepidermal Water Loss (TEWL) and Corneometer

TEWL (g/m^2^h) and hydration (arbitrary units, A.U.) in the stratum corneum (SC) were measured using a Tewameter (Courage Khazaka Electronic GmbH, Cologne, Germany) and a Corneometer^Ⓡ^ CM 825 (Courage Khazaka Electronic GmbH), respectively, at the end of experiment days. The measurement site was maintained at an indoor temperature of 22–24°C and a humidity of 50–60%. The measurement results were recorded three times (excluding the initial value), and the average value was determined.

### 2.8. RNA Preparation and Reverse Transcription-Quantitative Polymerase Chain Reaction (RT-qPCR)

At the end of the experiment days, the test animals were sacrificed; the lesion skin area was collected. Total RNA was extracted using TRIzol reagent (Invitrogen, Carlsbad, CA, USA). Synthesis of the first complementary DNA (cDNA) strand of the entire RNA template was performed using a Prime Script^TM^ RT Master Mix (Takara, Tokyo, Japan). The cDNA obtained was subjected to real-time PCR using qPCR 2X PreMIX SYBR (Enzynomics, Seoul, South Korea) and a CFX-96 thermocycler (Bio-Rad, Hercules, CA, USA). The PCR conditions used to amplify all genes were as follows: 30 cycles at 95°C for 10 min, 95°C for 10 s, 60°C for 15 s (IFN-*γ*, 53°C; IL-2, 54°C), and 72°C for 20 s. Expression data were calculated as cycle threshold (Ct) values using ΔCt through a quantification method. GAPDH was used for normalization. The oligonucleotides used are listed in Table 1.

### 2.9. Measurement of Serum IgE and Skin Thymic Stromal Lymphopoietin (TSLP)

At the end of the experiment, the test animals were sacrificed and whole blood and skin lesion area were collected; the blood sample were centrifuged at 3,000 rpm for 30 min at 4°C and the supernatant was collected. Proteins of skin were extracted using the PRO-PREP Protein Extraction Solution (iNtRON Biotechnology, Gyeonggi-do, South Korea). The total amount of IgE in the serum and TSLP in the skin were measured using an IgE Mouse Uncoated enzyme-linked immunosorbent assay (ELISA) kit (Invitrogen) and a TSLP rabbit Uncoated ELISA kit (Invitrogen) according to the manufacturer's instructions. IgE and TSLP expression were measured at a wavelength of 450 nm using a SpectraMax 190 microplate reader (Molecular Devices, LLC, Sunnyvale, CA, USA).

### 2.10. Statistical Analysis

Statistical significance was performed using GraphPad Prism version 7 software (GraphPad Software, San Diego, CA, USA). One-way analysis of variance and two-tailed unpaired *t* tests were performed. The experimental results are expressed as the mean ± standard error of the mean (SEM) and *p* values <0.05 were considered statistically significant. ^*∗*^*p* < 0.05, ^*∗∗*^*p* < 0.01 vs OXZ + Saline; ^#^*p* < 0.05, ^##^*p* < 0.01 vs Normal.

## 3. Results

### 3.1. Oral Administration of GTB1 Improves AD-like Lesion Symptoms

To investigate whether the oral administration of GTB1 was effective in improving AD symptoms, mice were sensitized with 5.0% OXZ on the back of the neck skin at -3 weeks, followed by challenges on the lower part of the back 1 week later with 0.3% oxazolone for 10 weeks (Figure 1(a)). No significant changes in weight were observed in the GTB1 oral administration groups compared to the saline group after 8 weeks, confirming that there were no safety issues associated with oral GTB1 administration (Figure 1(b)). The symptoms of AD were assessed by scoring excoriation, scaling, edema, and erythema (Figures 1(c) and 1(d)). The dermatitis score significantly increased to 11.70 ± 0.68 in the oral saline administration group as compared to the normal group (^##^*p* < 0.01). However, it significantly decreased to 8.60 ± 0.83 (^*∗*^*p* < 0.05) and 7.00 ± 1.20 (^*∗∗*^*p* < 0.01) in the OXZ + GTB1 7 and OXZ + GTB1 8 groups, respectively, as compared to OXZ + Saline group. In addition, the dermatitis score in the OXZ + 14917 group significantly decreased to 9.60 ± 0.64 (^*∗*^*p* < 0.05). Histologically, AD increases the thickness of the SC due to hyperkeratosis [22]. The epidermis thickness in the OXZ + Saline group was 83.83 ± 1.81 *μ*m, with a significant increase of 247.38% when compared with 24.13 ± 2.52 *μ*m in the normal group (^##^*p* < 0.01). Compared with that in the OXZ + Saline group, epidermal thickness significantly decreased by 29.74% and 36.30%, i.e., to 58.90 ± 2.46 *μ*m (^*∗∗*^*p* < 0.01) and 53.40 ± 5.60 *μ*m (^*∗∗*^*p* < 0.01), respectively, in the OXZ + GTB1 7 and GTB1 8 groups. In addition, epidermal thickness in the OXZ + 14917 group significantly decreased by 29.82% (to 58.83 ± 4.13 *μ*m; ^*∗∗*^*p* < 0.01) (Figures 1(e) and 1(f)).

### 3.2. Effects of GTB1 Oral Administration on Hypersensitivity Reaction

**Figure 2:**
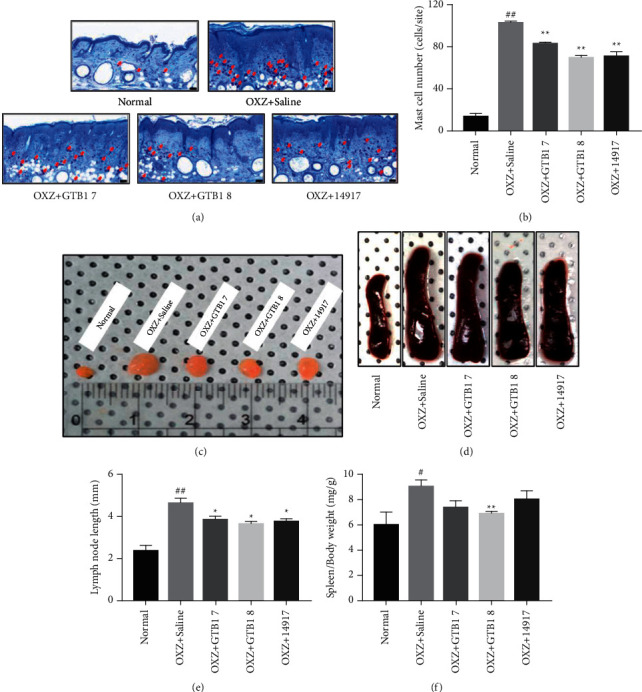
Histological examination of dorsal skin lesion using TB staining (20X, scale bar = 50 *μ*m) (a) (*n* = 3/group). Mast cell number (b) were measured by counting three spots in each group. Inguinal lymph node length (c, e) and spleen weight (d, f) were compared and measured after mice were sacrificed. OXZ + GTB1 7, GTB1 1 × 10^7^ CFU per mouse; OXZ + GTB1 8, GTB1 1 × 10^8^ CFU per mouse; OXZ + 14917, 14917 1 × 10^8^ CFU per mouse. Results are expressed as the mean ± SEM (*n* = 4/group). ^*∗*^*p* < 0.05, ^*∗∗*^*p* < 0.01 vs OXZ + Saline; ^#^*p* < 0.05, ^##^*p* < 0.01 vs Normal.

Mast cells are the main mediators of allergic inflammatory reactions and they secrete cytokines [23]. AD leads to increased mast cell infiltration at the sites of inflammation [24]. The number of mast cells significantly increased to 103.00 ± 1.15 in the OXZ + Saline group, compared to 13.00 ± 4.58 in the normal group (^##^*p* < 0.01). However, oral GTB1 administration led to a significant decrease in the number of mast cells in the OXZ + GTB1 7 and OXZ + GTB1 8 groups to 83.33 ± 1.33 (^*∗∗*^*p* < 0.01) and 70.33 ± 1.76 (^*∗∗*^*p* < 0.01), respectively, compared to the OXZ + Saline group. In addition, the number of mast cells in the OXZ + 14917 group was also reduced to 71.67 ± 3.84 (^*∗∗*^*p* < 0.01) (Figures 2(a) and 2(b)). A common feature of chronic inflammation is enlargement of immune organs including spleen and lymph nodes [25,26]. Compared with those in the normal group, inguinal lymph node size and spleen/body weight significantly increased by 94.74% (^##^*p* < 0.01) and 51.27% (^#^*p* < 0.05), respectively, in the OXZ + Saline group. Inguinal lymph node size in the OXZ + GTB1 7 and OXZ + GTB1 8 groups significantly decreased by 16.22% and 21.62%, respectively, in a CFU-dependent manner (^*∗*^*p* < 0.05), when compared with those in saline group. In addition, we observed a significant decrease to 18.92% in the OXZ + 14917 group (^*∗*^*p* < 0.05) (Figures 2(c), 2(e)). Spleen/body weight was found to significantly decrease by 24.66% in the OXZ + GTB1 8 group alone when compared with that in the OXZ + Saline group (^*∗∗*^*p* < 0.01) (Figures 2(d), 2(f)). This result indicates that GTB1 can attenuate the inflammatory reactions by OXZ.

### 3.3. Oral GTB1 Administration Restores Epidermal Skin Barrier Function

**Figure 3:**
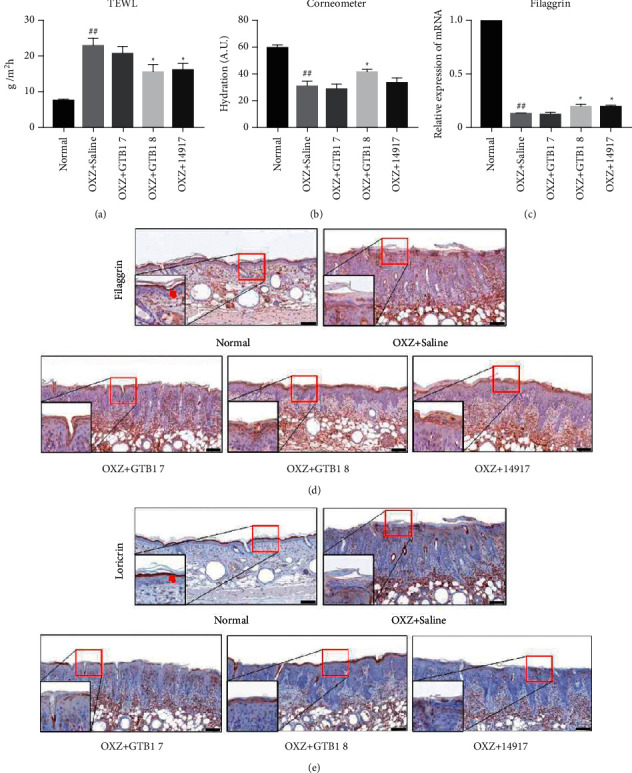
At end of the experiment, TEWL (a), and hydration (b) levels in the AD-like mouse skin lesions were measured (*n* = 5/group). Skin tissue filaggrin mRNA expression levels was determined by RT-qPCR analysis (c) (*n* = 3/group). DAB staining for filaggrin (d) and loricrin (e) expression (15X, Scale bar = 100 *μ*m). OXZ + GTB1 7, GTB1 1 × 10^7^ CFU per mouse; OXZ + GTB1 8, GTB1 1 × 10^8^ CFU per mouse; OXZ + 14917, 14917 1 × 10^8^ CFU per mouse. Results are expressed as the mean ± SEM. ^*∗*^*p* < 0.05 vs OXZ + Saline; ^##^*p* < 0.01 vs Normal.

AD is the most common chronic skin disease and have increased TEWL. The saline group with AD-like lesions observed that TEWL significantly increased while SC (stratum corneum) hydration decreased. However, oral administration of GTB1 8 decreased TEWL (^*∗*^*p* < 0.05) (Figure 3(a)) and increased hydration levels (^*∗*^*p* < 0.05) (Figure 3(b)). Skin barrier dysfunction in AD leads to pathogen invasion and subsequent immunological responses. Filaggrin is an essential protein for the correct formation and function of the skin barrier [27]. The filaggrin expression is downregulated in AD patients, likely as a downstream effect of T helper cells type 2-derived (Th2) cytokines such as interleukin (IL-) 4 and IL-13 [28]. To evaluate the effect of GTB1 on the epidermal skin barrier, the expression of filaggrin was measured.

The expression of filaggrin was measured. The filaggrin mRNA expression was significantly increased in the OXZ + GTB1 8 and OXZ + 14917 groups than in the OXZ + Saline group (^*∗*^*p* < 0.05) (Figure 3(c)). The OXZ + Saline group showed lower filaggrin and loricrin expression levels than the normal group. The GTB1 oral administration group increase the expression of filaggrin and loricrin in a CFU-dependent manner compared to the OXZ + Saline group. The 14917 oral administration group also showed an increase in the levels of these proteins that constitute the skin barrier (Figures 3(d), 3(e)). These results suggested that GTB1 could restore the damage of skin barrier by OXZ.

### 3.4. Oral GTB1 Administration Regulated the Th2/Th1 Immune Response

**Figure 4:**
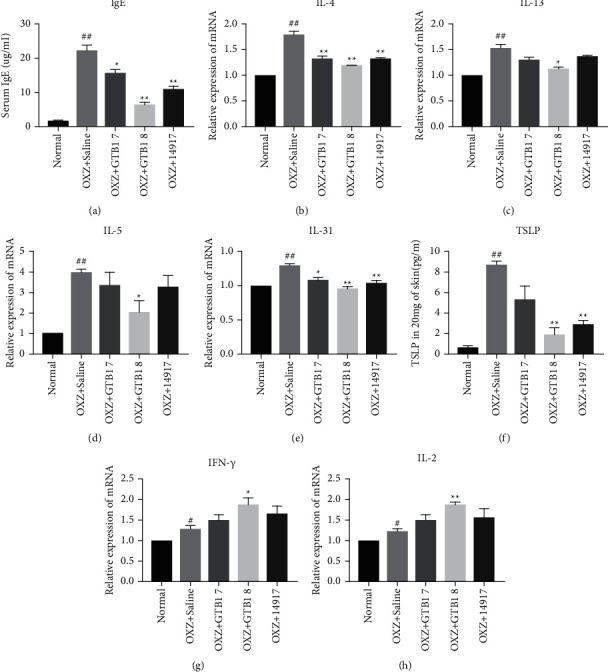
At the end of the experiment, skin tissue and serum were collected. Total serum IgE levels were determined by ELISA (a). The relative IL-4 (b) and IL-13 (c), IL-5 (d), and IL-31 (e) mRNA expression levels in skin tissue were determined by RT-qPCR analysis. Skin tissue TSLP levels were determined by ELISA (f). The relative IFN-*γ* (g) and IL-2 (h) mRNA expression levels in skin tissue were determined by RT-qPCR analysis. OXZ + GTB1 7, GTB1 1 × 10^7^ CFU per mouse; OXZ + GTB1 8, GTB1 1 × 10^8^ CFU per mouse; OXZ + 14917, 14917 1 × 10^8^ CFU per mouse. Results are expressed as the mean ± SEM (*n* = 3/group). ^*∗*^*p* < 0.05, ^*∗∗*^*p* < 0.01 vs OXZ + Saline; ^#^*p* < 0.05, ^##^*p* < 0.01 vs Normal.

IgE plays a key role in the degranulation of the mast cells [24, 29]. The serum IgE expression level was significantly decreased in all probiotic groups when compared with that in the OXZ + Saline group (Figure 4(a)). Th1 and Th2 cell specific cytokines expression were analyzed using RT-qPCR and ELISA analysis at the end of the experiment. Levels of IL-4, which is a type of Th2 cytokine, were significantly decreased in all probiotic groups when compared with those in the OXZ + Saline group (Figure 4(b)). IL-13 levels significantly decreased only in the OXZ + GTB1 8 group as compared to the OXZ + Saline group (^*∗*^*p* < 0.05) (Figure 4(c)). The expression of IL-5, which is associated with dermal eosinophil infiltration, and those of IL-31 and TSLP factors, which is associated with itching, was significantly decreased in the OXZ + GTB1 8 group (Figures 4(d)–4(f)). Furthermore, the expression of IFN-*γ* and IL-2, which are Th1 cytokines, significantly increased only in the OXZ + GTB1 8 group (Figures 4(g), 4(h)). Our findings support anti-inflammatory effects of GTB1 on allergen-specific immune responses.

## 4. Discussion

In the present study, the inhibitory effects of GTB1 were evaluated in an OXZ-induced AD-like lesion mouse model. Although various methods are available to establish AD models, the hapten method is usually applied. This is because hapten do not elicit an immune response by themselves as they are usually <1 kDa in size and need to bind to carrier proteins to elicit an immune response [30]. Man et al. [20] previously reported that OXZ, a type of hapten, induced contact dermatitis in mice, induced Th2-like hypersensitivity reactions, increased IgE expression levels, and decreased barrier function, similar to AD. Therefore, the OXZ-induced AD-like lesion model used in this study is a suitable model for investigating the inhibitory effects of AD.

AD is a common clinical manifestation that involves 2 major biological pathways: skin barrier abnormalities and immune dysfunction. This study investigated the protective effect of GTB1 on AD-like symptoms in skin barrier and immunological aspects. The main symptoms of AD are excoriation, scaling, edema, and erythema [31]. In the groups receiving oral GTB1, the dermatitis score decreased after 8 weeks in a CFU-dependent manner (Figure 1). Loricrin is a component of the outer cell membrane and filaggrin is the connective and cohesive keratin intermediate microfibers within the outer cell membrane and is decomposed into amino acids, hydrocarboxylic acids and trans-urocanic acids which are associated with natural moisturizing factors [28, 32]. When filaggrin decreased due to AD, it leads to dryness in the SC, reduction of the overall skin barrier function, and increases both external allergen penetration and TSLP expression resulted in create a Th2-dominant cytokine environment and skin barrier abnormalities [33, 34]. Filaggrin and loricrin expression levels were increased by oral GTB1 administration (Figure 3). These results indicate that GTB1 has the potential to alleviate AD symptoms.

IL-4 and IL-13 are the most important factors in AD pathogenesis and treatment, and these factors induce IgE expression through class switching in B cells [35, 36]. TSLP is highly expressed in keratinocytes during AD and stimulates the secretion of IL-4, IL-5, and IL-13 [37] and causes pain and itching [38]. After oral GTB1 administration, the mast cell numbers (Figure 2) and serum IgE levels were decreased. The IL-4 and IL-13 mRNA expression levels were decreased. Moreover, the levels of IL-5 and IL-31 mRNA expression and TSLP protein were decreased (Figure 4). Taken all together, GTB1 can suppress the AD mediated-inflammatory response. The gut microbiome is an important contributing factor to the immunologic pathway of AD via probiotics. According to the gut-skin axis theory, oral administration probiotics interact with epithelial cells, mucosal dendritic cells (DCs), and macrophages through diverse ways [19]. Through these interactions, probiotics contribute to improving allergic hypersensitivity reactions such as AD by balancing Th2/Th1 immune responses [39–41]. We confirmed that the oral administration of GTB1 increases the expression level of IFN-*γ* and IL-2, which are Th1 cytokines (Figure 4). Recently, the dysbiosis in gut microbiota is one of the factors of the cause of AD [42]. Furthermore, we have already confirmed that GTB1 has an antibacterial effect on *Staphylococcus aureus*, known as the most common pathogen in skin and gut microbiome in AD patients [43]. Taken all together, oral GTB1 administration has the possibility to treat the AD. However, this study has some limitations. First, the small number of mice in each group has limited statistical power in describing the significant differences. Second, the efficacy of oral GTB1 was not compared with that of conventional AD treatments, such as systemic steroids, topical steroids treatment, and antihistamines. Therefore, future studies should compare the efficacy of conventional AD treatments and oral GTB1 administration. Overall, GTB1 has potential as a supplementary medicine for the improvement of AD.

## 5. Conclusions

In summary, the present study demonstrated that GTB1 isolated from green tea (*Camellia sinensis*) improved the clinical symptoms, including dermatitis score, epidermis thickness. In addition, GTB1 reduces mast cell number, IgE level, lymph node size, and spleen weight. GTB1 also increase filaggrin and loricrin level. Finally, it is involved in AD improvement by regulating the Th2/Th1 cytokine balance. These results suggest that novel GTB1 could serve as a potential supplementary medicine for the improvement of AD.

## Figures and Tables

**Table 1:** Oligonucleotides used for RT-qPCR.

Gene name	Forward (5′⟶3′)	Reverse (5′⟶3′)
IL-2	AGATGAACTTGGACCTCTGC	TGGCACTCAAATGTGTTGTC
IFN-*γ*	CACACTGCATCTTGGCTTTG	TCCACATCTATGCCACTTGAG
IL-4	GGTCTCAACCCCCAGCTAGT	GCCGATGATCTCTCTCAAGTGAT
IL-5	CTCTGTTGACAAGCAATGAGACG	TCTTCAGTATGTCTAGCCCCTG
IL-13	CCTGGCTCTTGCTTGCCTT	GGTCTTGTGTGATGTTGCTCA
IL-31	TCAGCAGACGAATCAATACAGC	TCGCTCAACACTTTGACTTTCT
Filaggrin	ATGTCCGCTCTCCTGGAAAG	TGGATTCTTCAAGACTGCCTGTA
GAPDH	AGGTCGGTGTGAACGGATTTG	TGTAGACCATGTAGTTGAGGTCA

## Data Availability

All data generated or analyzed during this study are included in this published article.
